# From biomedical cloud platforms to microservices: next steps in FAIR data and analysis

**DOI:** 10.1038/s41597-022-01619-5

**Published:** 2022-09-08

**Authors:** Nathan C. Sheffield, Vivien R. Bonazzi, Philip E. Bourne, Tony Burdett, Timothy Clark, Robert L. Grossman, Ola Spjuth, Andrew D. Yates

**Affiliations:** 1 Center for Public Health Genomics, School of Medicine, University of Virginia, 22908 Charlottesville, VA USA; 2 School of Data Science, University of Virginia, Charlottesville VA 22904, Charlottesville, VA USA; 3 Department of Biomedical Engineering, School of Medicine, University of Virginia, 22904 Charlottesville, VA USA; 4 Department of Public Health Sciences, School of Medicine, University of Virginia, 22908 Charlottesville, VA USA; 5 Department of Biochemistry and Molecular Genetics, School of Medicine, University of Virginia, 22908 Charlottesville, VA USA; 6 Deloitte, 20201 Baltimore, MD USA; 7 European Molecular Biology Laboratory, European Bioinformatics Institute, Wellcome Genome Campus, Hinxton, Cambridge, CB10 1SD UK; 8 Center for Translational Data Science, University of Chicago, Chicago, IL 60615 USA; 9 Department of Pharmaceutical Biosciences and Science for Life Laboratory, Uppsala University, 75124 Uppsala, Sweden

**Keywords:** Computational platforms and environments, Standards, Hardware and infrastructure

## Abstract

The biomedical research community is investing heavily in biomedical cloud platforms. Cloud computing holds great promise for addressing challenges with big data and ensuring reproducibility in biology. However, despite their advantages, cloud platforms in and of themselves do not automatically support FAIRness. The global push to develop biomedical cloud platforms has led to new challenges, including platform lock-in, difficulty integrating across platforms, and duplicated effort for both users and developers. Here, we argue that these difficulties are systemic and emerge from incentives that encourage development effort on self-sufficient platforms and data repositories instead of interoperable microservices. We argue that many of these issues would be alleviated by prioritizing microservices and access to modular data in smaller chunks or summarized form. We propose that emphasizing modularity and interoperability would lead to a more powerful Unix-like ecosystem of web services for biomedical analysis and data retrieval. We challenge funders, developers, and researchers to support a vision to improve interoperability through microservices as the next generation of cloud-based bioinformatics.

## The rise of cloud platforms

**Fig. 1:**
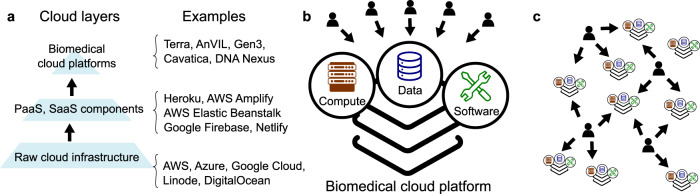
Biomedical cloud platforms. (**a**) Biomedical cloud platforms are built from generic cloud components, including raw infrastructure components and Platform-as-a-Service (PaaS) or Software-as-a-Service (SaaS) offerings from commercial or on-premises providers. (**b**) A biomedical cloud platform provides compute power, data, and software for a particular purpose, and provides efficiency as a single source where users can come to run analysis. (**c**) Biomedical cloud platforms have proliferated, leading to a bewildering array of offerings and confusion for biomedical scientists.

Cloud computing skyrocketed in popularity between 2008–2010 with great promise. Now, as cloud infrastructure has matured, a more recent trend in bioinformatics is the rapid expansion of biomedical cloud platforms and related “commons” sharing environments. We define biomedical cloud platforms as computing environments that provide three things: (1) access to specific (biomedical) data resources; (2) specific biomedical analysis software; and (3) cloud compute power for analysis. Biomedical cloud platforms are virtual environments specialized for a use case or research community. They are built using general-purpose cloud components or services, such as commercial Infrastructure-as-a-Service or Platform-as-a-Service products, but provide specificity to a biomedical research area in the form of tailored data and tools (Fig. 1a).

In bioinformatics, cloud platforms and related frameworks are expanding rapidly; to name a few, there is Terra, AnVIL^1^, the Gabriella Miller Kids First Data Resource Center^2^, Cavatica^3^, Gen3 Workspaces^4^, the Biomedical Research Hub^5^, the AHA Precision Medicine Platform^6^, CanDIG^7^, NHLBI BioData Catalyst, Alex’s Lemonade Stand Refine.bio, the Pediatric Cancer Data Commons^8^, SAGE Bionetworks’ Synapse^9^, the NCI Genomic Data Commons^10^, and others. In addition, commercial providers such as DNA Nexus, Seven Bridges, and Truwl offer custom platforms.

It’s clear that the biomedical research community is investing heavily in the concept of cloud platforms, and there are several compelling arguments for this^11^. The most oft-cited argument revolves around the idea that for big data, it is more efficient to bring analysis to data than vice versa^1^. Clearly, as biological data has grown, it has become harder to move around. Transfer times and costs are substantial, and even if the data can be transferred, many researchers don’t have sufficient local resources; and for those that do, it feels terribly inefficient to duplicate petabytes of data, particularly since most of the time these datasets are just sitting around. Cloud platforms seem like the perfect solution: Put all the data in one place, and let the researchers come and analyze it there in a controlled computing environment (Fig. 1b).

Besides potentially reduced costs for data storage and transfer, cloud platforms provide other advantages for all datasets, whether large or small: they allow computing over a dataset with less friction. For example, cloud platforms simplify controlling access to data, which is critical for many types of biological data. They also enable centralized, standardized computing environments, which have the potential to make analysis more reproducible while simultaneously reducing the need for investigators to maintain their own computing infrastructure. Users can start with a notebook with all required libraries, tools, and applications already loaded, sidestepping challenges like software compatibility, network architecture, server maintenance, etc. This is the experience that users have come to expect from platforms like Kaggle and Colab. Finally, biomedical cloud platforms offer easy and virtually unlimited scalability for both storage and compute power, with “pay-as-you-go” pricing that is favorable to the bursting use case common in biomedical research. These advantages lend great appeal to the biomedical cloud platform model, driving enormous investment over the past few years.

And yet somehow, despite large investments in biomedical cloud platforms, the promise of a unifying cloud seems further away than ever. Funders, companies, research institutions, and even individual lab groups have begun setting up their own biomedical cloud platforms, leading to a bewildering array of offerings. Each is optimized for a particular purpose and comes with data and analytical resources specific for that task. But conflictingly, in a pitch to win users, these same platforms are often marketed as flexible and accommodating, and encourage researchers to bring their own tools and data to bear within the ecosystem. In a way, although the explosion of cloud platforms reflects their varying tailored use cases, they nonetheless seem to be chasing the “holy grail” of the one cloud platform to rule them all. Paradoxically, instead of consolidating computing environments per the original intent, investment in biomedical cloud platforms has actually increased the number of places to go and things to learn for biomedical data analysis (Fig. 1c).

If a single computing environment could accommodate all a researcher’s computing needs, this wouldn’t necessarily be a problem; however, researchers typically work on multiple projects with differing tool or data requirements that cannot necessarily be fulfilled by the same platform. Their needs may span different large datasets, include private data that cannot leave the premesis, or include specific hardware requirements. Thus, rather than reducing the challenges of computing and big data for individual labs, researchers are now divided even further as they must learn to use local high-performance computing environments mixed with multiple cloud environments, each with its own idiosyncrasies. This cloud platform heterogeneity is even more intense than that of the underlying cloud infrastructure providers; indeed, biomedical cloud platforms also spread across clouds, leading to duplicated administrative burden like managing billing accounts, learning user interfaces, and understanding provider-specific terminology. Despite the sound rationale and good intentions of cloud platforms, they have not simplified computing on big data, but increased its complexity.

Perhaps these biomedical cloud platform woes are just growing pains as the community grapples with new ideas and technology. As we gain experience, perhaps the task of managing data across platforms will become more manageable. Maybe competition among them will lead to consolidation and simplification. If so, over time, these struggles will be mitigated, and biomedical cloud platforms may eventually achieve the promise of simplifying and democratizing biomedical data analysis. But is it possible that these issues are not transient, and instead reflect an inevitable emergent property of the platform approach?

## Cloud platforms inherently prioritize internal features

**Fig. 2:**
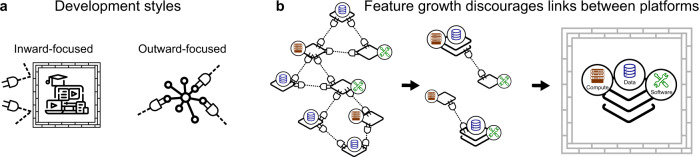
Inward-facing platform development decreases interoperability. (**a**) Biomedical cloud platform development is often inward-focused, which prioritizes building capabilities of the platform. In contrast, outward-focused development emphasizes connections to other platforms. (**b**) As platforms develop new capability, they have less need to connect to other platforms, eventually leading to a self-contained environment.

In our experience, biomedical cloud platforms, while frequently built on the premise of simplifying data sharing and interoperability, are not generally delivering on this promise. Instead, as we argue further below, they are leading to lock-in, more restricted data access, non-interoperable data schemas, and duplicated effort for users and developers. One reason for this is the appeal of a common design choice: they tend to prioritize internal platform capabilities over external connections that drive interoperability with other systems (Fig. 2a). We define internal development as work on standalone capabilities of the platform itself; things like breadth of available analysis tools, amount of hosted data, and flexibility with types of analysis or workflows that can be run on the platform. Internal capabilities represent the utility of a platform and are obviously necessary. However, too much focus on internal capabilities neglects external connections–that is, links to resources outside the platform, such as the ability to retrieve and integrate data from another cloud platform, or send data to an analysis tool hosted elsewhere.

The drive to prioritize internal features over external ones has several natural causes: First, developers often find it easier or more rewarding to develop their own systems than to investigate others; second, funders generally prioritize biomedical outcomes and consider external connections of secondary interest; third, user counts are often considered a measure of success for funding and prestige, disincentivizing developers from connecting to competitors; fourth, platform architects generally make decisions independently and do not consider the emergent issues in the community as many people make the same decisions. These factors lead to cloud platforms that tend to grow in provided features. As the platforms expand in scope and power, the incentive to invest in interoperability across platforms decreases, because more and more external functions become available internally. This creates a self-reinforcing cycle in which individual platforms begin to sell simplicity in the form of a system to solve all the problems for the researcher in-house, so that the researcher doesn’t need to go anywhere else to get things done. The premise is ultimately to enable researchers to do work–any work at all–inside the platform, with all necessary data at hand.

These promises are appealing, but while the intention behind the approach for each individual platform may seem justifiable and desirable, the incentives and practical limitations means in practice, cloud platforms often end up as walled gardens (Fig. 2b).

## Biomedical cloud platforms lead to practical challenges

The emergent properties of an ecosystem of dozens or hundreds of internally-focused platforms lead to several practical issues: (1) platform lock-in; (2) difficulty integrating across platform technology; (3) tight coupling around *ad hoc* data schemas; and (4) confusion and duplicated effort for both users and developers.

### Platform lock-in

A major challenge with the biomedical cloud platform approach is the difficulty switching platforms due to both financial and cultural barriers. On the financial side, major cloud providers price storage to make it easy to get data in, but hard to get it out. This business decision makes it difficult to leave a particular cloud. Although this may not be an intentional property of a biomedical cloud platform, it is often inherited from the underlying provider. Finances aside, there is also a cultural cost in switching platforms. To become familiar with a particular cloud platform requires a substantial learning investment; each comes with its own terminology, focus, and idiosyncrasies. This learning cost must be repaid when switching platforms, making it a chore. These financial and cultural costs mean once a dataset or research group commits to a particular cloud platform, it is difficult to change. The bigger the data gets, the more inertia there is to just leave it there. This is a negative quality in a fast-moving scientific enterprise.

### Difficulty integrating across platforms

The thought of a single biomedical cloud platform for all your work is very appealing; but unfortunately, this simplifies the process of real research, particularly in biology. More and more, analysis requires integrating data from many sources, both public and private. Furthermore, the types of analyses are also frequently split across many kinds of tools with different computing needs. This heterogeneity increases the complexity of a one-size-fits-all cloud platform. And even if a particular platform can satisfy all needs for a particular project, most researchers are working on multiple projects, each with a different set of reference data, private data, and analysis tools. Furthermore, the dream of a central analysis location is a myth in the first place: though we may envision cloud infrastructure as a nebulous, remote environment in the sky, the reality is that cloud infrastructure, too, has a physical location and is necessarily split across regions and clusters. These issues together make it inevitable that researchers will need to integrate across cloud platforms. The inward-facing focus of cloud platforms ignores this complexity, assuming instead that the researcher will be able to complete every project from start-to-finish using only the resources within a single cloud platform.

### Tight coupling around ad hoc data schemas

Cloud platforms may be offered as a more FAIR alternative to self-hosted research data. This argument arises because self-hosted data often adopt ad-hoc or project-specific standards, whereas cloud platforms have an opportunity to emphasize community-accepted data standards. However, the real driver of interoperability comes not from cloud platforms *per se*, but from community data schemas they may promote. Unfortunately, the incentives that drive inward-focused features also lead platforms to adopt project-specific data standards in the same way as self-hosted data. Cloud platforms, in their pursuit of more data and more users, are incentivized to be agnostic to standards and formats that support findability and reuse, and instead promote accessibility that encourages analysis in place and discourages downstream sharing. This creates an illusion of FAIRness: putting terabytes of data into one large BigQuery table does not make that data interoperable. Instead, it pushes ever greater costs for collecting, cleaning, and harmonizing data onto data generators and data scientists who already cite this as their biggest challenge. Thus, bespoke but cloud-native tools with tightly-coupled *ad hoc* schemas portents a new generation of project-specific data silos that are much more expensive to combine and clean.

### Confusion and duplicated effort for both users and developers

Users must investigate and select the platform that fits best for their question. Given the fast-moving area of cloud computing and the large number of available offerings, coupled with the cost of learning the systems due to their complexity, plus the importance of the decision due to the difficulty to switch–this is a substantial decision that users should not take lightly. If they do need to switch, this cost will need to be paid repeatedly. The other major cost is developer time. Under the cloud platform model, many common needs will need to be re-implemented independently in each platform.

These practical challenges share a theme: data interoperability. Interoperability is one of the four core tenets in the FAIR Data Principles^12^, which are community-accepted guidelines for data management created to drive greater uptake and reuse of data assets^13–19^. FAIR places very specific interoperability requirements on data; for example: (1) data must be identified with persistent globally unique identifiers with two-stage resolution; (2) resources must be described using rich metadata that is searchable; (3) metadata must be represented in a formal vocabulary and accessible separately from the data; etc.^12,19–23^. The FAIRness concept is also being expanded to software^24,25^.

Pursuing FAIRness is a difficult journey, and one that many researchers and organizations are just beginning. Data interoperability is generally the hardest of the four principles to adopt, and our four practical challenges above demonstrate some of the difficulties. We contend that these interoperability issues are not simply growing pains, but an inevitable corollary that emerges with many independent decisions to develop standalone, inward-focused cloud platforms. Because the current incentive structure encourages inward-facing development over outward-facing connections, our effort is leading to lack of interoperability, data silos, and increased complexity for both users and developers. Enormous investments in cloud platforms are not only not helping, but may even be hurting our ability to share and analyze data.

But if cloud platforms aren’t the way forward, what is?

## Interoperability through microservices

We propose to encourage data interoperability by shifting effort from biomedical cloud platforms to microservices. The term microservices encompasses multiple concepts that together form an architectural style for application development. The *micro* part means individual components are small and individually useful. The *services* part means execution of the function is remote, and therefore doesn’t rely on local computing infrastructure. Thus, microservices are typically small functions, executed by a remote service, often via API. The microservice approach breaks an application into a series of loosely coupled functions that together accomplish a complex process.

Microservices can be viewed either as the antithesis, an extension, or a foundation of cloud platforms. Where biomedical cloud platforms derive their appeal from the breadth of internal data and features they provide, microservices provide few capabilities and derive power from interoperability. Therefore, unlike monolithic platforms, microservices development naturally leads to increased interoperability. Microservices are a modern-age, web-based rediscovery of the Unix philosophy: “[T]he power of a system comes more from the relationships among programs than from the programs themselves. Many UNIX programs do quite trivial tasks in isolation, but, combined with other programs, become general and useful tools”^26^. After nearly 5 decades, Unix-like systems have become widely used for web services and now dominate the list of top supercomputers^27^, a testament to the enduring legacy of the Unix philosophy.

But are microservices a practical possibility in the extremely complicated and heterogeneous world of biological data analysis, in which large, complex datasets and resource-intensive, multifaceted analysis are the norm? On the surface, the idea of small services and small data retrieval inherent to microservices seems at odds with common challenges in biological data analysis. How can the benefits of microservices apply to huge data sets spread across multiple data providers? How are microservices relevant when long-running analysis tasks are required to process the data?

Though the complexity and scale of biomedical analysis do make it more challenging to reap the benefits of microservice-based development, we propose that key concepts from microservices can still be very useful for application in biological data analysis. We propose two concepts for improving interoperability of large-scale centralized data: (1) data slicing; and (2) data summary layers.

### Data slicing

**Fig. 3:**
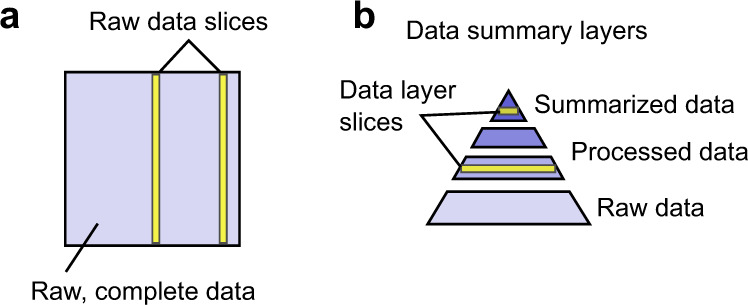
Granular data access approaches improve interoperability. (**a**) Data slice APIs provide access to data in chunks, rather than to the entire dataset at once. (**b**) Data summary layers provide granular access to data at multiple stages of processing.

From a platform-centric perspective, we build platforms around data because it’s more efficient to bring analysis to data than the inverse. Taken to its extreme, the most efficient and convenient analysis platform would be a single unified platform hosting all possible data. Unfortunately, this is impractical; there will always be multiple data sources in multiple locations, and therefore data transfer is inevitable for any analysis that spans data sources. Nevertheless, most of the time, most of the data is at rest, and for many analyses, only small chunks of data from several large data stores are needed. A microservices-centric perspective posits an alternative solution: to provide easy access to data slices (Fig. 3a). A microservice for data slicing provides efficient API-oriented access to small chunks of data. Like centralizing all data, data slicing also solves the problem of moving analysis to data, but in a different way: by encouraging the data to move, but minimizing the amount of data that must do so. By making it easy to move the data in small parts, data slicing microservices make cross-dataset analysis practical and efficient–without requiring an impossible single platform that hosts all data.

### Data summary layers

Access to data slices is useful for analyses that only require specific subsets of a large dataset, but what about tools or analyses for which slices of data do not suffice? Some analyses, such as machine learning applications, require looking across complete data sets. While there may be no simple solution for analysis that demands all raw data, many tools in this category, including common machine learning methods, benefit from summarized, cleaned, and compressed data. This leads to a second approach to improve data interoperability: data summary layers (Fig. 3b). In this approach, we conceptualize data as moving through a process that progressively reduces the size: raw data is large, processed data is much smaller, and summary data is very small. A machine learning application typically consumes processed or summarized data that has already been simplified and normalized–for example, by extracting the relevant features for a particular application. Another example is an API providing access to data via a trained model, which is in itself a form of summarized data. By providing access to data in summarized or processed form, a service minimizes the amount of transfer similar to data slices, but this time by providing a processed version of the complete data set.

Providing access to data slices or summaries leads to several benefits. First, it mitigates some regulatory concerns on data privacy; raw data can be controlled while allowing unfettered access to summarized or sliced pieces of data, which in many contexts is allowed. Even the most restricted datasets have some summary level that can be made widely available, and for access levels that require authorization, granular access is easy to implement using modern microservice-based authorization frameworks like OAuth 2.0. Second, it improves the ability to share data, which increases interoperability among tools and analyses by encouraging tools to use the same loosely connected set of data sources. Third, it improves reproducibility and reusability, because a particular data chunk can be reproducibly obtained from the server using a static API URI. Fourth, it simplifies the analytical setup for the user: Instead of providing yet another computing environment so researchers can bring analysis to data, it allows researchers to use the computing environment they are already comfortable with. Finally, by increasing emphasis on interoperability, this approach will also emphasize standards creation and uptake.

## The rising tide of microservice-friendly technology

Comparing monolithic cloud platforms against microservices is a resurfacing of a perennial question in application development: to what degree should a tool rely on external libraries or remote services versus implementing them in-house? The same question arises in web development, software engineering, Linux containers, etc. Outsourcing has the advantage of eliminating duplication, reducing size and maintenance, and increasing interoperability; however, it requires reliable outsourced functions. If an external library or service is not reliable, robustness is improved by embedding capability internally. This is particularly true in web applications, where services may be down not only for software reasons, but also due to lack of network stability. Unfortunately, for an ecosystem built around microservices, a single unreliable service may cause an entire pipeline to stop.

Reliability is therefore a justifiable argument against microservices. However, at least from the technological point of view, it is rapidly being addressed. As cloud computing has matured, the technology and stability of the Internet has progressed to where it is no longer a dream to build reliable and fault-tolerant applications that are distributed across services and servers; in fact, it is quickly becoming appreciated that such an approach is likely to yield more stable systems in the long run because it simplifies scaling, troubleshooting, and redundancy. In the past, technological and network issues may have been a strong argument for building self-contained cloud platforms, but today, technology has risen to a point that the opposite is now true, and the most robust applications are typically built from distributed service components that operate and scale independently.

Other recent technological advances are also making the microservice-based approach more amenable in terms of maintainability and ease of development. Microservice implementations are facilitated by containerization and orchestration frameworks such as Kubernetes^28^. While these systems have previously been demanding to set up and hence constituted a barrier, they are now straightforward to contextualize on virtually every cloud infrastructure. Further, most bioinformatics software is available in containers, a trend that is simplifying their incorporation in microservices and microservice-based platforms^29^.

Microservices and containerized software support not only web application development, but also bioinformatic pipelines that process data through a sequence of compute steps. For example, pipelines can use microservices as sources of reference data, to outsource compute tasks, or to share or publish processed data. In contrast, a pipeline built into a monolithic, inward-focused cloud environment is more likely to rely on internal reference data and tools, and is thus more tightly coupled to a particular platform with tools and reference data not necessarily available for use by other pipelines in other environments for other purposes. Microservices therefore improve the portability and interoperability of pipelines.

## Existing work in bio-microservices

The explosion in biomedical cloud platforms doesn’t mean there has been no effort to build bio-related microservices; on the contrary, many applications adhering to microservice principles are under development and in use. For example, efforts across the Global Alliance for Genomics and Health (GA4GH) are driving a variety of microservices, such as Refget^30,31^. The Gen3 Framework Services are a small set of microservices that have been used as a foundational set of services to develop over a dozen different data commons and data resources, each developed independently for a different research community to support their individual requirements^5^, but all relying on the same set of core microservices. Another example is the PhenoMeNal project, which pioneered Virtual Research Environments (VREs) for metabolomics based on the microservice architecture, with many microservices in metabolomics analysis and general data analysis^32,33^. The OpenRiskNet project (openrisknet.org) refined this environment for chemical safety assessment and added an API layer for semantic discoverability for services. The NCI Genomic Data Commons provides API-oriented access to petabytes of genomic data, which can also be accessed in data slices^10^. Other efforts include refgenie^34,35^, FAIRSCAPE^36^, and others. Tools like these are already an indispensable part of the ecosystem of biological computing. However, because microservices by nature have small scope, they typically get neither the public recognition nor funding given to monolithic efforts.

## The next generation of microservice-enabled cloud platforms

**Fig. 4:**
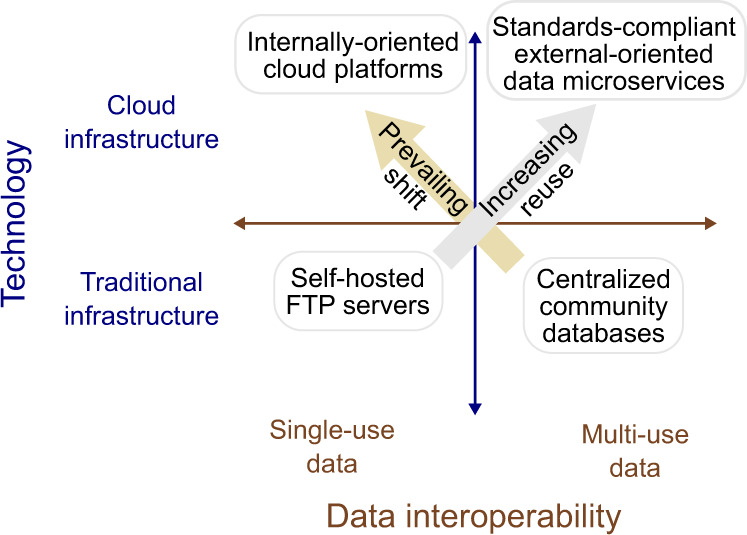
Interoperability will require attention to both technology and data. Infrastructure technology is orthogonal to data interoperability. Emphasis on cloud technology alone may ignore data reuse. Considering both technology and data reuse could usher in a new era of data interoperability.

We have argued that emphasis on biomedical cloud platforms leads to emergent challenges with interoperability. But the problem is not with platforms *per se*, but rather, with the common practice of emphasizing inward-facing capability at the cost of interoperability. The prevailing trends are encouraging inward-facing capability, rather than standards-compliant, external-facing services (Fig. 4). In fact, a cloud platform built from accessible services^37^ could provide the best of both worlds: a unified location for specific analysis with specific data resources, plus an offering of interoperable services to retrieve small data or run small tasks from diverse external environments. This concept is similar to the previously proposed idea of data commons, defined as a cloud platform that also provides access to data via an API^38^. For example, the NCI Genomic Data Commons provides a series of API services, and then builds additional capability on top of these services. If the microservices are made first-class components accessible externally, this development approach could lead to a robust, Unix-like interoperable system of services that may be mixed and matched.

To encourage further development of the vision for an interoperable network of microservices, we offer several recommendations for the community: First, we challenge developers of cloud platforms to focus more effort on APIs, even if this comes at the cost of internal platform feature development. These APIs should follow existing guidelines, including being OpenAPI-compatible^39^. Furthermore, APIs should not just provide access to data, but should provide granular access to chunks and summarized forms of data. Second, we propose that effort should shift from platforms that provide computing services toward platforms focused on data services. The primary goals of a data platform should be (1) to process the data to a summarized form in a reproducible way; (2) to make that data easily accessible, in chunks and summaries, so that users can reliably and reproducibly retrieve the subset or summarized version of the data they need. Only as a secondary goal should platforms provide the ability to bring compute power to work on the raw data. This vision inverts the common practices today in cloud platforms. Third, we challenge funding bodies to increase investment and sustained support of small, focused services, rather than large, self-contained and all-encompassing platforms. One way to do this is through organizations like GA4GH, which are large enough to command attention and drive use, but divided into small work streams that tackle small, isolated problems and produce interoperable microservices. Another way to do this would be to develop smaller, sustained grant mechanisms that provide for maintenance and development of well-defined microservices. Fourth, we recommend increasing emphasis on coordination activities in general. Coordination across both large consortia and individual groups is resource-intensive, but critical for generating interoperable datasets and tools. Coordination effort adds tremendous scientific value, so increased funding and recognition of these activities will promote open standard and data interoperability. Finally, we appeal to grant and paper reviewers to prioritize interoperability and granularity of data access of a platform, rather than just scope of features. From our experience, there is a disturbing trend in the review process that encourages revisions of grants and papers to increase impact by broadening scope and size, which drives effort away from development of microservices. This effort could instead be invested in polishing interoperability. Proposals should be evaluated rigorously against published FAIR principles^12^ for any funded biomedical commons environment or framework.

We are convinced that emphasizing microservices will lead to improved interoperability in biomedical data analysis. However, other approaches and further work are clearly necessary; achieving interoperability has proven one of the most challenging aspects of modern data analysis, and it will require a variety of technical and cultural adjustments to get right. Interoperability has many levels^20^; for example, interoperability of underlying data and software sets the stage for interoperability among cloud platforms and frameworks, which in turn encourages interoperability among downstream analytical results. While microservices can solve some technical challenges, their benefit will be limited if the related issue of interoperable data schemas is not addressed. Furthermore, technical interoperability as discussed here is just the beginning, and is only useful if the underlying data itself is consistent. For example, different experimental approaches may yield data that are difficult or impossible to integrate, regardless of FAIRness. Nevertheless, improving the technical interoperability, including more machine-friendly metadata description, is an important universal step that can set the stage for substantive coalescence of experimental protocols and analysis methods that can make the data not only technically but also biologically interoperable. Furthermore, computing technology under development have potential to further enhance biomedical data interoperability. For example, distributed computing, edge computing, and content delivery networks (CDNs), are driving performance and challenging centralized computing models. Serverless computing and serverless databases are reshaping the data landscape. Microtransactions have become a viable business model and may provide a solution to the incessant challenge of “who pays?” in biomedical computing. Developments like webassembly are increasing the appeal of local compute. These innovations and others all have potential in a community effort to improve interoperability. Together, we are convinced that with the effort of developers, funders, reviewers, and users, we can realize a more interoperable ecosystem for biomedical data analysis.
